# Communicating as a physician-scientist

**DOI:** 10.1172/JCI167652

**Published:** 2022-12-15

**Authors:** Elizabeth McNally

**Affiliations:** Center for Genetic Medicine, Northwestern University Feinberg School of Medicine, Chicago, Illinois, USA.

In early March 2020, a time that seems like yesterday and also like a century ago, we grasped the immediacy of the global pandemic arriving to our shores as spring breakers returned to campuses with COVID-19 and universities responded. As educators, scientists, and physicians, we adapted to remote learning and telehealth, and work in our laboratories slowed or halted. The annual meeting of the three organizations that make up the Joint Meeting, APSA, ASCI and AAP, was suspended.

Over the spring and into early summer 2020, we were unsure what to expect and specifically what the world might look like over the next year. In spring of 2020, two of my graduate students defended their theses. They held virtual dissertation defenses, and we had no in-person gatherings to celebrate their accomplishments. One student left for her postdoctoral fellowship, and the other student returned to medical school to begin his rotations in the middle of a pandemic and advance his physician-scientist training.

In late 2020, as the pandemic ravaged early autumn and took us into a dark, cold winter, I talked frequently with my student who had transitioned from his dissertation work to his rotations on the wards to finish medical training. He told me he didn’t think he would ever be a doctor without wearing a mask. His words transported me back in time to when I was a medical student, when the HIV epidemic engulfed New York City, and the concept of “universal precautions” became the norm. Universal precautions meant wearing gloves routinely for all patients, not just those we knew had infectious diseases. Then with HIV and now with SARS-CoV-2, we know our tests are limited — both because of the technical nature of molecular testing as well as the biological nature of the disease — we knew there were those who spread the viral infection without knowing they were infected.

Throughout 2020, in my hometown of Chicago, we had additional challenges. The murder of George Floyd at the hands of police in Minneapolis set Chicago ablaze several times throughout the summer of 2020, leading to true lockdowns. With these lockdowns, there was no transit into large parts of Chicago’s downtown area — not by car, not by public transportation — and we experienced extended curfews. In August 2020, an hour from Chicago, the shooting of Jacob Blake and its aftermath, including an AR-15–armed teenager, left us frightened and shaken.

But then in very late 2020, early data emerged from vaccine trials, and it showed vaccines were effective at preventing hospitalization and death. This gave us so much hope, and I received my first mRNA vaccination on Christmas Eve 2020. We watched the nation vaccinate, and we saw hospitalizations and deaths diminish. Despite this progress, we still did not feel we could gather safely as the Joint Meeting in 2021, and this time, with a bit more runway, we held the first virtual Joint Meeting, which was so ably orchestrated by our prior presidents of ASCI, AAP, and APSA.

The omicron tsunami hit hard in December 2021, and it threatened the 2022 Joint Meeting. Despite this threat, in April 2022, we held an in-person meeting for the first time in three years. The 2022 Joint Meeting had amazing science, but among the most inspirational of talks was from Dr. Katalin Kariko with her emphasis on persistence. It was this persistence that enabled new vaccine technology, saving millions of lives and allowing our health care systems to function. These vaccines, born of decades of fundamental research, have been transformational. Despite the highly validated scientific method that produced this progress, many of us, me included, were surprised to encounter mistrust of new technology and especially for vaccines, masks, and medications. Medical mistrust is not new, but medical mistrust has exponentially grown, goaded by a widening spread of antiintellectualism and antielitism.

As a practicing physician, I find mistrust is more commonplace than I have ever before experienced. I am especially concerned by physician-scientists who promote unsubstantiated ideas and claims and who intentionally fan the flames of suspicion and conspiracy. During this pandemic, certain physician-scientists vastly overstepped their knowledge and expertise, even promoting ideas that are not in the interest of human health. Some pushed false beliefs about “herd immunity” of populations who were “protected” and did not need vaccines. As a community of scientifically oriented practitioners, we hold a responsibility to counter these claims and to correct those who promote these falsehoods.

For better and sometimes for worse, the pandemic has meant we have had to communicate to the public much more about science. Mostly this has been for better. The public now has a greater appreciation of so many aspects of research and medicine — much of the lay public understands, in a general sense, what a virus is and what having antibodies means. They have a better grasp of molecules, including RNA, and even of the challenges of clinical investigation. They have learned about observational science and the importance of local data and local conditions. Many have come to know our public health officials by name and face. The public increasingly appreciated that the US public health system is fragmented and not federated. And we also learned that we each approach data in different ways, bringing our backgrounds and biases and sometimes just seeing what we want to see.

My scientific background requires me to consider DNA sequences, molecular structures, chromatin conformation, and how these structures come together inside of cells. I collaborate with population and data scientists as much as with laboratory-based researchers. I remain fascinated by the rare and unique variation found in each genome and how even a single, individual base pair change can have devastating and profound outcomes on health. These rare pathological genetic variants drive our lab’s research questions and also guide my clinical interests because these variants cause disease and, in many cases, profoundly disabling disease.

I am a practicing physician, and this very much influences how I view data. I provide care to patients with genetic disorders, including some genetic disorders that render their heart, muscles, and, importantly, their respiratory muscles weakened. The pandemic has been very frightening for so many people, including my patients; their breathing is compromised, and their hearts are often failing, and many would not leave their homes. Through telehealth, we learned to address many needs, including fears and personal questions about how to best manage their conditions and their health. Across a broad educational range and background, many patients were reasonably informed throughout the pandemic. However, in visits, my patients did not want to hear about population data; they wanted to hear about what they should do. They didn’t want to hear about case fatality rates; they wanted to know whether they would die. They wanted to know what mask they should wear and whether they could go to a restaurant.

Some patients expressed their fear of COVID vaccines. The fear and hesitance were not explained by any one thing. Some expressed reluctance about new technology, testing, and government involvement in vaccine development, but many just expressed a mistrust that I had not seen before. This mistrust was also in patients I have known for years. They just didn’t trust these vaccines, and they didn’t believe what I had to say about them. Despite the historically unparalleled success of vaccine development and implementation, I heard many misconceptions from my patients and, even harder, I heard misconceptions from my own family members.

I heard the belief that vaccines are dangerous and killing people. One vaccine-hesitant patient told me that older people he knew received vaccines and then would be hospitalized with new diagnoses like cancer. It is these mistaken “cause and effect” relationships that propel conspiracy theories, and conspiracy theories are much more common than we may want to believe. Patients may be reluctant to tell us about their mistrust; they may nod along, but they may not really agree.

The group of patients that simply don’t believe in medical and scientific knowledge has grown. Or more likely, patients may pick and choose which parts of medicine may fit their belief system. Increasingly, this selective patient mistrust may have political associations or derive from nontraditional sources of information. The sources of misinformation and even disinformation are many, and we, as a community of physician-scientists, need to do more to combat disinformation.

Dissemination of disinformation is partly attributed to curated news feeds that propagate echo chambers. Artificial intelligence–driven algorithms of page views, likes, and dislikes engender a self-affirming sense of shared misbeliefs. These “sources” of authoritative information include professional athletes “doing their own research,” and they also include politically motivated doctors and scientists. We underestimate how much misinformed sources have replaced our voices. As we incorporate social determinants of health in research and health care delivery, we should address health illiteracy as a major social determinant of health. Health disinformation contributes to health illiteracy, and dismantling disinformation requires counter measures.

We need to do more, and we should look closely at medical communication to the public — where it has worked well and where it can do better. Physicians and scientists are now frequently wearing the hat of “public communicator” on local and national news and extensively in social media. Many from the community of physician-scientist leaders are already effective communicators, but that does not mean we cannot improve. Here are steps that can help.

## Admitting when we do not know

We can start by acknowledging that we have never lived through this before. The world has never experienced a pandemic at this scale accompanied by the current capabilities and expectations of modern technology and medicine. It is inherently flawed to draw conclusions from prior pandemics, especially from influenza a century ago. Coronaviruses are different; they spread and mutate in patterns distinct from other viral subtypes. We also track viral spread now using a plethora of new technology, from individual rapid tests to waste-water sampling — methods we did not have previously. For those who say “pandemics last two years” based on influenza in 1917–1919, we need to remember we have not been through this before. These viruses are not the same, and the world is not the same. We need to always articulate the extent of our knowledge and admit that knowledge rapidly changes.

## Acknowledge the limitations of observational science

We need to be clear with the public when inferences derive from observational data. As an example, multiple reports have “confirmed” SARS-CoV-2 came from a natural source and not a lab leak. The certainty of these findings was reinforced on the front page of the New York Times, but with a failure to acknowledge the observational nature of the data. We know the virus readily transmits between humans and animals, and we also know that the world was granted limited access to data from early 2020 in Wuhan.

Observational data must always be carefully discussed, highlighting where the data may be wrong or biased, whether the topic is case fatality rates or untested treatments. Assertions based on observational data can often be biased and nonrepresentative. In sports, there are statistical analytics for nearly every imaginable competitive situation, yet the games are still played, and the predictions often fail.

## Be truthful and avoid loaded words

With 2020 hindsight, it is common to hear laments over “lockdowns” with physician-scientists asserting “millions of people died from lockdowns.” “Lockdown” is a politically loaded and inaccurate term especially when used to describe the US. In the US, we were not locked in our houses, and the persistent use of this term extends misinformation.

## Explain why advice may shift over time

The vacillating early advice on masking has often been cited as proof of the ineffectiveness of masking. Very early in the pandemic, masking was not recommended, and the harsh reality of the early pandemic was that here in the US, we did not have masks since they are manufactured internationally. Domestically, we still do not adequately manufacture personal protective equipment, and it remains a weak link. Schools have always been sites of infectious disease spread; children share germs and then spread germs in the household. Had we had enough masks and been willing to use them, there may have been no need to close schools in 2020.

## When you speak as a physician, you are heard as a physician

When physicians speak, they are heard as doctors. Much of the public views public statements from physicians through the lens of the doctor-patient relationship. When we speak as physicians and scientists, we are heard as physicians, and we should be more cognizant of this distinction. Those who use observational data to address population trends are not providing individualized recommendations. My patients expect me to know the population trends and then advise what is best for them knowing them as individuals.

Our communications to patients and the public should respect our science and medical expertise, but statements should be tailored to appreciate the nuances of speaking as a physician. Some physicians have expertise in areas beyond medical care, including economics, law, and policy. But this additional expertise often is obscured when one is introduced as a physician. If you are introduced as a physician, you are likely to be heard as a physician.

## When we speak as doctors, we cannot violate the public trust

Talking to the public is not the same as talking to peers. It is normal for peers to debate scientific ideas. Peer review and debate can often lead to more carefully stated, softened interpretations. When planning to speak publicly, consider asking a peer, perhaps even a less like-minded peer, to review what you want to say before you say it.

What we can do as physician-scientists is use our training and knowledge to provide more accurate assessment of the data at hand. It is our responsibility to call out bad data and those who push it. We should point out the flaws, especially if the data are not representative or incorrectly interpreted. This is where we can lead and teach.

To build and rebuild trust will take time and effort. We bring a unique perspective when we communicate as physician-scientists. We see the science, and we also see the potential to change lives. We can communicate our excitement about a breakthrough to the public, but there are times when our exuberance for what is possible may get the better of us, and we should resist overpromising. Good scientists know that discovery, especially fundamental laboratory-based, empirical science, moves forward in fits and starts, often with more days taking steps backwards than taking steps forward. Part of our duties as physician-scientists is to communicate to the public, but this should be in the same context of the oaths we took when we became physicians. Just as there is a doctor-patient relationship, there is a doctor-public relationship, and in this relationship, we should embrace our same ideals and we should limit harm.

As we develop better guidance about our roles as public educators, we will also need to grapple with how to manage those among us who promote faulty interpretations. This is not the time to look away when we hear physicians speaking in a manner detrimental to public and individual health. This is the time to address misinformation in a frank and deliberate manner. We hold this responsibility as part of our mission to improve human health, and this means being willing to point out those who fail to meet these expectations.

## Version 1 12/15/2022

Electronic publication

